# Expression Analysis of Stress-Related Genes in Kernels of Different Maize (*Zea mays* L.) Inbred Lines with Different Resistance to Aflatoxin Contamination

**DOI:** 10.3390/toxins3060538

**Published:** 2011-06-09

**Authors:** Tingbo Jiang, Boru Zhou, Meng Luo, Hamed K. Abbas, Robert Kemerait, Robert Dewey Lee, Brian T. Scully, Baozhu Guo

**Affiliations:** 1 Department of Plant Pathology, The University of Georgia, Tifton, GA 31793, USA; Email: tjiang@uga.edu (T.J.); zhouboruha@hotmail.com (B.Z.); Kemerait@uga.edu (R.K.); 2 Key Laboratory of Forest Tree Genetic Improvement and Biotechnology of Ministry of Education, Northeast Forestry University, Harbin 150040, China; 3 Department of Crop and Soil Sciences, The University of Georgia, Tifton, GA 31793, USA; Email: mluo@agcenter.lsu.edu (M.L.); deweylee@uga.edu (R.D.L.); 4 Department of Plant Pathology and Crop Physiology, Louisiana State University, Baton Rouge, LA 70803, USA; 5 United States Department of Agriculture-Agriculture Research Service, Biological Control of Pests Research Unit, Stoneville, MS 38776, USA; Email: hamed.abbas@ars.usda.gov; 6 United States Department of Agriculture-Agriculture Research Service, Crop Protection and Management Research Unit, Tifton, GA 31793, USA; Email: Brian.Scully@ars.usda.gov

**Keywords:** resistance genes, gene expression, qPCR, aflatoxin contamination

## Abstract

This research examined the expression patterns of 94 stress-related genes in seven maize inbred lines with differential expressions of resistance to aflatoxin contamination. The objective was to develop a set of genes/probes associated with resistance to *A. flavus* and/or aflatoxin contamination. Ninety four genes were selected from previous gene expression studies with abiotic stress to test the differential expression in maize lines, A638, B73, Lo964, Lo1016, Mo17, Mp313E, and Tex6, using real-time RT-PCR. Based on the relative-expression levels, the seven maize inbred lines clustered into two different groups. One group included B73, Lo1016 and Mo17, which had higher levels of aflatoxin contamination and lower levels of overall gene expression. The second group which included Tex6, Mp313E, Lo964 and A638 had lower levels of aflatoxin contamination and higher overall levels of gene expressions. A total of six “cross-talking” genes were identified between the two groups, which are highly expressed in the resistant Group 2 but down-regulated in susceptible Group 1. When further subjected to drought stress, Tex6 expressed more genes up-regulated and B73 has fewer genes up-regulated. The transcript patterns and interactions measured in these experiments indicate that the resistant mechanism is an interconnected process involving many gene products and transcriptional regulators, as well as various host interactions with environmental factors, particularly, drought and high temperature.

## 1. Introduction

The fungal metabolite aflatoxin is among the most potent naturally occurring carcinogens, and is produced primarily by *Aspergillus flavus*. Aflatoxin contamination has been a chronic problem in maize (*Zea mays* L.) production in the Southern U.S. for many decades. Warm, humid conditions favor growth of the *A. flavus* fungus resulting in severe ear rot, while hot, dry weather favors high aflatoxin production. Breeding for resistance, or more accurately kernel and plant characteristics that inhibit infection by *Aspergillus* ear rot and aflatoxin production, is currently considered the most desirable means of controlling aflatoxin production [1]. Identification and/or development of host resistance is the most widely explored strategy for eliminating or reducing aflatoxin contamination, and germplasm screening studies have identified a number of inbreds and breeding lines, such as Tex6 and Mp313E [2,3,4]. More basic genetic research is needed to explain the maize resistance mechanisms within various biochemical pathways, and based on molecular functionality and gene expression [5]. It is generally concluded that resistance to aflatoxin in maize kernels is a multigenic quantitative trait with a large genotype x environment interaction [6]. 

Maize crops are often exposed to many abiotic and biotic stresses, and some stress-related proteins have been reported to not only confer stress-tolerance, but also enhance resistance to diseases and aflatoxin contamination [7,8]. Proteomic comparisons have identified many stress-related proteins along with antifungal proteins associated with kernel resistance [9,10]. We analyzed the expression levels of 94 stress-related genes in seven maize lines with different levels of susceptibility to *A. flavus* infection and aflatoxin contamination in order to better understand the gene expression pattern in kernels of these lines as well as the aflatoxin levels. Therefore, the objectives of this research were to compare the expression levels of stress related genes in susceptible and resistant maize lines under well watered and drought condition and to develop a set of genes/probes associated with resistance to *A. flavus* and/or aflatoxin contamination. These candidate genes are available for further examination across a diverse set of inbreds [11].

## 2. Materials and Methods

### 2.1. Plant Materials

Maize inbred lines: B73, Lo1016, Mo17, Mp313E, A638, Tex6, and Lo964 were grown in the field along with two controls, GTP2 and GTP27 [2], at Belflower Farm, Tifton, GA, USA, in a Tifton loamy sand soil. Peanut and corn were previously rotated biannually. The field trials were designed as a randomized complete block with 6 replications for aflatoxin analysis. Experiment plots were 6.0 m long and spaced 0.76 m apart with 2.4-m alleys. The ear shoots were bagged before silk emergence, and ears were self-pollinated. The pinbar method was used for the inoculation with *A. flavus* spores at 21 days after pollination (DAP). Inoculated ears were hand harvested at maturity for aflatoxin analysis with ELISA and HPLC methods as described by Abbas *et al.* [12]. 

To enhance gene expression analysis, the seven inbred lines were grown in field rain-out shelters with clear plastic cover with drought stress imposed by moving the shelter over the plots at V5 stage. Ears were self-pollinated, drought stress conditions were then initiated by the cessation of irrigation at 25 DAP in the rain-out shelters while normal irrigation continued in control shelters. The intensity of drought stress was monitored by measuring photosynthesis efficiency of the leaf at or near the top ear. Ear samples were collected at 35 DAP. Analyses of three sub-samples per replicate were performed for each sample. Immediately upon collection samples were frozen in liquid nitrogen and subsequently stored at −80 °C until analysis.

### 2.2. Gene Expression Analysis Using qPCR

In the previous work [1,13], 299 kernel stress related genes with known function were discovered by microarray analysis, and 94 of these genes were selected for further scrutiny (Table 1). Gene expression levels among different maize germplam were analyzed by qRT-PCR. Total RNA was extracted from kernels of a single ear of each inbred line selected from harvested samples of each drought stress treatment using TRIzol reagent (Invitrogen, Carlsbad, CA, USA) and performed in accordance with the manufacturer’s instructions. Isolated total RNA was then treated with DNase (Qiagen, Valencia, CA, USA) and purified using an RNeasy Cleanup Kit (Qiagen). Purified total RNA was then checked for quality and quantity using a Nano-Drop ND-1000 Spectrophotometer (Thermo Scientific, Wilmington, DE, USA).

**Table 1:** Expression levels of 94 stress-related genes in different maize lines.

Gene ID	Putative Annotation	B73	Lo1016	Mo17	Mp313E	Tex6	Lo964	A638	B73 *	Tex6 *
TC273692	heat shock protein 21	--	--	-	+	++	+	++	××	++
TC248621	early drought induced protein	--	--	--	++	++	++	+	+	+
TC247852	abscisic acid inducible gene	--	--	--	++	++	+	++	+	--
TC260723	putative salt-inducible protein kinase	-	-	-	+	+	+	+	××	-
TC249150	proline-rich protein family-like	--	--	-	+	++	+	++	+	-
TC261320	MAP kinase phosphatase	-	--	-	+	+	+	++	++	-
TC248521	lipid transfer protein	++	--	--	++	++	++	++	--	+
TC259179	γ-thionin	-	--	+	++	++	++	+	××	+
TC270625	glutathione reductase	-	--	××	+	++	+	+	-	××
TC248890	putative glutathione peroxidase	++	--	--	++	++	++	++	-	-
TC252272	multi resistance protein	+	--	--	++	++	+	+	××	--
TC260617	putative MAP kinase	-	--	××	+	+	+	+	××	++
TC261534	putative hydroxyproline-rich glycoprotein DZ-HRGP	××	--	+	++	+	+	++	+	++
TC261606	leucine-rich repeat transmembrane protein kinase	××	--	-	+	+	+	+	++	+
TC248912	dehydrin DHN1	+	++	++	++	--	++	××	+	++
TC258769	LEA14-A	++	++	++	++	--	++	--	++	++
TC269764	glyoxalase I	-	--	-	-	++	+	++	+	-
TC263499	diamide resistance gene	-	--	-	××	++	+	+	+	++
TC248251	putative stress-related protein	--	--	--	++	-	++	++	++	+
TC271560	heat shock protein hsp22 precursor	-	--	-	-	++	-	++	++	++
TC273584	oxidation protection protein	--	--	-	××	+	+	++	+	++
TC248631	unknown (myoD protein inhibitor)	-	--	-	××	+	××	++	-	++
TC271775	mitogen activated protein kinase	-	-	-	××	+	××	+	-	++
TC272055	putative HSPC058	-	-	-	-	+	+	++	××	-
TC250756	polyphenol oxidase	-	-	-	--	++	++	++	+	++
TC249851	multidrug-resistance associated protein	-	--	-	××	+	+	+	+	++
TC248721	N/A	--	--	--	++	-	-	++	++	+
TC260636	leucine-rich repeat resistance protein-like protein	--	-	-	+	+	-	+	-	+
AZM4-134720	similar to water stress inducible protein	--	--	--	××	++	-	++	--	+
TC263714	major facilitator superfamily antiporter	-	-	--	+	++	+	××	--	+
TC262308	putative glycolate oxidase	--	--	-	++	+	-	++	-	+
TC259689	cysteine protease	--	--	-	++	++	-	++	+	--
TC261493	thionin like protein	-	--	--	××	+	++	++	+	××
TC251520	alpha globulin	-	--	-	××	-	+	++	+	-
TC259802	putative stress-induced protein	-	--	-	+	+	+	××	-	--
TC258326	L-ascorbate peroxidase	-	--	-	××	+	++	++	++	--
TC271380	probable trypsin inhibitor	--	--	--	++	++	--	++	--	××
TC268744	putative hydroxyproline-rich glycoprotein 1	--	--	--	++	++	++	--	--	+
TC250985	unknown protein	--	-	-	-	××	++	++	--	+
TC269707	r40g2 protein	--	--	-	+	++	-	+	+	--
TC272484	putative UVB-resistance protein	--	--	--	-	++	+	++	-	+
TC261400	receptor protein kinase PERK1-like protein	--	--	--	++	++	--	++	--	++
TC251180	hybrid proline-rich protein	××	++	++	--	++	++	--	-	+
TC258497	metallothionein-like protein	-	--	+	××	++	××	+	-	-
TC270445	γ-zeathionin 1	-	--	++	--	++	++	++	+	+
TC248731	chitinase	--	-	××	--	++	++	++	+	+
CF630432	bet v I allergen	-	-	××	+	××	+	+	-	+
TC269763	subtilisin/chymotrypsin inhibitor	--	+	++	××	--	++	+	-	-
TC272650	putative stress-inducible membrane pore protein	--	--	××	++	+	-	++	××	+
TC262243	expressed protein	-	+	++	--	++	+	-	--	+
TC260600	peroxiredoxin	--	-	++	--	++	++	××	××	-
TC248296	nonspecific lipid-transfer protein precursor	--	-	++	--	++	++	++	+	××
TC271062	NAM-related protein 1	--	--	+	-	-	++	++	-	++
TC260324	putative xylanase inhibitor protein	--	++	++	--	-	++	++	+	+
TC251880	metallothionein	××	+	--	++	++	++	-	+	--
TC270149	globulin-1S	--	--	××	××	+	++	++	+	-
TC253617	putative serine/threonine-specific protein kinase	--	-	+	+	-	+	++	--	++
TC253449	late embryogenesis abundant protein	--	++	++	++	--	++	++	+	++
TC261509	putative aldose reductase	-	-	+	--	--	++	++	++	++
TC273536	heat shock protein	+	-	-	--	++	++	××	++	+
CF000577	proline rich protein	--	××	++	--	--	++	++	++	++
TC259915	hageman factor inhibitor	+	--	××	++	--	+	+	+	--
TC270070	putative universal stress protein	××	--	××	××	××	+	++	++	--
TC258155	putative glutathione S-transferase	--	+	++	+	--	++	++	-	++
TC270514	hypothetical protein(aluminum-induced protein-like)	++	-	--	+	-	-	++	-	+
TC248921	putative peroxisome type ascorbate peroxidase	--	+	××	++	+	××	++	-	++
TC249614	Superoxide dismutase [Mn]	-	××	-	+	+	+	-	--	++
TC258876	hydroxyproline-rich glycoprotein precursor	××	-	+	+	--	+	+	-	++
TC270339	superoxide dismutase 2	++	--	++	++	--	+	++	-	++
AI372267	peroxidase	++	+	--	-	++	--	--	++	-
TC259921	antimicrobial peptide	××	-	+	--	++	++	+	+	-
TC270782	putative leucine-rich repeat transmembrane protein kinase	+	--	--	++	++	--	++	--	××
TC271639	putative cytochrome P450	××	++	--	++	++	--	++	--	++
TC259396	catalase	--	-	+	++	××	+	××	--	++
TC249070	Superoxide dismutase [Cu-Zn]	+	-	--	++	+	+	-	+	-
TC271423	glutathione S-transferase	--	××	--	++	++	++	-	+	+
TC270868	globulin 2	--	--	××	-	+	++	++	××	--
TC259180	γ-zeathionin 2	-	--	+	+	++	++	-	-	--
TC268733	s-adenosylmethionine synthetase	-	-	××	+	++	××	-	--	--
TC270842	cold shock protein	+	--	-	+	++	××	××	+	××
TC260707	salt-induced AAA-Type ATPase	--	-	××	+	××	+	++	++	+
TC250578	putative beta-1,3-glucanase	+	--	--	++	+	--	+	--	++
TC268849	heat shock protein	++	--	--	--	--	-	++	++	++
AI372246	lipoxygenase	+	--	--	--	+	++	++	-	++
TC251457	putative proline-rich protein	--	--	++	--	--	-	++	+	++
TC260910	putative CC-NBS-LRR resistance protein MLA13	--	++	--	××	××	++	++	++	++
TC264819	putative antifungal zeamatin-like protein	--	--	++	-	-	--	++	++	++
TC261221	chalcone synthase	--	--	××	××	--	+	++	--	++
TC249478	ascorbate peroxidase	××	--	--	××	++	××	++	++	××
TC259722	cysteine protease component of protease-inhibitor complex	++	--	++	--	++	++	-	--	++
TC202729	ribosomal inactivating protein	××	--	--	+	++	-	++	--	××
TC263586	antifungal zeamatin-like protein	+	--	--	++	++	-	++	++	--
AZM4-123774	phenylalanine ammonia-lyase	+	-	+	-	++	-	+	+	--
TC273961	flavanone 3-hydroxylase	--	--	++	--	+	-	++	--	++

Expression level of each gene was obtained from the real-time qPCR analysis using the 2^−ΔCT^ method and expressed as relative expression of the gene of interest and the reference gene; and “+” used to show up-regulated between 1.1 to 2 folds, “++” for up-regulated over 2 folds, “-” for down-regulated 0.5 to 0.9 time, “--“ for down-regulated under 0.5 time, and “××” as unchanged. Lines with “*” indicated the gene expression under drought versus well-watered condition.

One-step qPCR was performed using a QuantiTect SYBR Green RT-PCR kit (Qiagen) on DNA Engine Opticon Continuous Fluorescence Detection System (MJ Research, Inc.) according to the manufacturer’s instructions. A total reaction volume of 25 µL containing 300 ng total RNA and 25 µM of each primer was used in this study. The expression level of maize glyceraldehyde-3-phosphate dehydrogenase (GAPDH, Accession No: U45856), amplified with primer pair G-F (5′-ACTGTTCATGCCATCACTGC-3′) and G-R (5′-GAGGACAGGAAGCACTTTGC-3′), was used as an internal control.

Amplification curves were generated from the real-time qPCR data and the cycle threshold (C_T_) was calculated based on a fluorescence threshold of 0.01, where C_T_ was defined as the threshold cycle of PCR at which an amplified product was first detected. Subsequently, the ΔC_T_ for each sample was determined using the equation ΔC_T_ = C_T_ target gene—C_T_ reference gene to calculate the relative expression of each gene to the internal reference control. This was accomplished via a modification of the original equation to relative expression = 2^−ΔCt^ for both the control and treatment samples [14,15]. The hierarchical cluster of maize inbred lines based gene expression level was performed using Gene Cluster 3.0, and the graphical representation of the tree was obtained by TreeView 1.60.

## 3. Results

### 3.1. Aflatoxin Contamination in Different Maize Lines

Previous research has indicated that Tex6 and Mp313E consistently exhibit resistance to aflatoxin contamination, while Mo17 and B73 were susceptible to aflatoxin contamination [3,4]; Lo964 is drought tolerant with a very intensive root system, while Lo1016 is drought susceptible with a superficial and extensive root system [16], and previously unknown for their aflatoxin reaction; A638 showed a higher resistance to stalk rot in contrast to B73 [17] and had drought tolerance. Aflatoxin concentrations in these inoculated lines ranged from 291 ppb to 964 ppb (Table 1); Tex6, Lo964 and Mp313E exhibited relatively lower levels of aflatoxin contamination, whereas B73, Lo1016, A638 and Mo17 exhibited relatively higher aflatoxin contamination (Table 2). The two controls, GTP2 and GTP27, had higher levels of aflatoxin but GTP27 had aflatoxin levels similar to Mo17. In other studies, GTP27 was consistently susceptible to *Aspergillus* infection and higher aflatoxin contamination [2].

**Table 2:** Field evaluation of different maize inbred lines for aflatoxin contamination

Genotypes	Aflatoxin (ppb) ^a^	Significance	Reference^ b^	
GTP2	924 ± 455	a		
B73	737 ± 45	a	1557	271
Lo1016	663 ± 136	ab		
A638	560 ± 192	ab		
Mo17	507 ± 108	ab	950	152
GTP27	494 ± 134	ab		
Mp313E	434 ± 71	b	34	
Lo964	332 ± 125	bc		
Tex6	291 ± 136	c	586	39

^a^ Six replications in the field, mean ± SE within columns followed by the same letter did not differ significantly (*P* > 0.05). ^b^ Aflatoxin levels as reported [3,4].

### 3.2. Differential Expression of Stress-Related Genes in Different Maize Lines

**Figure 1:**
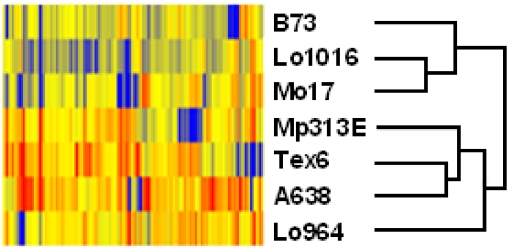
Hierarchical clustering analysis of 94 gene expression data in different maize inbred lines. Samples with similar patterns of expression of the genes clustered together. The average level of the gene expression among seven inbred lines in this study was used as the control. Red indicates up-regulation, blue indicates down-regulation, and yellow indicates unchanged.

**Figure 2:**
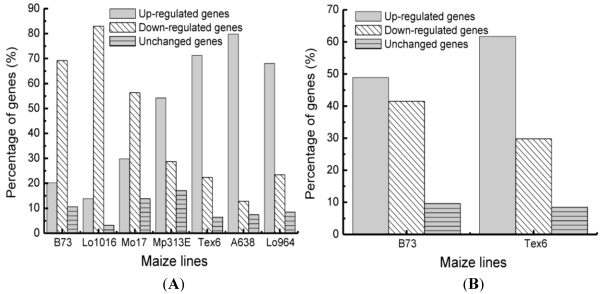
Percentage of genes expressed in different patterns using qRT-PCR. (**A**) The average level of the gene expression in all seven lines was used as control; (**B**) shows the differential expressed gene under drought condition *versus* well-watered condition.

The expression levels of the 94 stress-related genes among seven maize inbred lines revealed the diversity patterns of gene expression in different lines. The same gene was expressed differently in the tested lines, and each maize inbred line had differential patterns of different gene expressions (Table 1). Based on the relative-expression levels of the 94 genes, the inbred lines clustered into two different groups (Figure 1), which correlated well with the aflatoxin levels (Table 2). Group 1 (susceptible group) includes B73, Lo1016 and Mo17, and Group 2 (resistant group) included low-aflatoxin Tex6, Mp313E, Lo964 and A638. By comparing the expression of these 94 stress-relative genes in these two groups, there were more genes expressed higher than average (up-regulated genes) in the resistant Group 2 maize lines with lower-aflatoxin than in the susceptible Group 1 maize lines with higher-aflatoxin (Table 1 and Figure 2). In contrast, there were more genes expressed lower (down-regulated) in the susceptible Group 1 than in the resistant Group 2 (Figure 2). 

**Figure 3:**
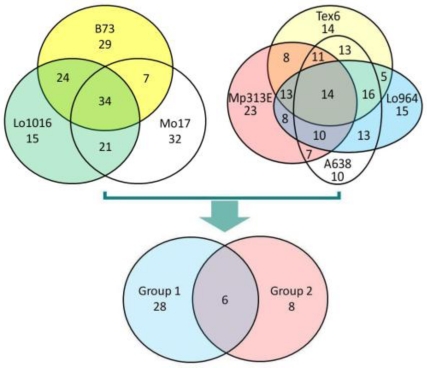
Possible cross-talking genes in different maize inbred lines. The grouping was done based on the same expression patterns (up-, down, or unchanged). The expression patterns of the 6 common genes in both groups were different, down regulated in Group 1 (susceptible group) and up regulated in Group 2 (resistant group).

By comparison of the expression pattern (up-regulated, down-regulated, or unchanged) of each gene in different maize lines, there were a total of 34 genes with the same expression patterns in the susceptible Group 1 and 14 genes in the resistant Group 2 (Table 1, Figure 3). However, there were only six genes expressed differently in these two groups (Table 1, Figure 3): down-regulated in the susceptible Group 1 and up-regulated in the resistant group 2. The thirty-four genes in Group 1 included two genes with higher expression, dehydrin DHN1 (TC248912) and LEA14-A (TC258769), but these two genes were expressed differently in Group 2 (Table 1). The 14 genes in Group 2 were all highly expressed in all maize lines in this resistant group.

During drought stress 49% and 62% of the 94 genes were up-regulated in B73 and Tex6, respectively, while 42% and 30% were down-regulated in B73 and Tex6 (Figure 2). However, there were 31 genes with same expressed patterns in both lines, including 25 up-regulated genes and six down-regulated genes. This study supported the previous studies reporting that B73 and Tex6 were different in resistance to drought stress and aflatoxin contamination [1,6,13].

## 4. Discussion

The expressions of stress and/or defense related genes were significant and play an important role in regulatory mechanisms in developing protection of the kernels in drought stress. Luo *et al.* [1] reported that gene expression in response to drought stress in Tex6 kernels may happen when stress reaches an acute level. For instance, drought stress was introduced at 18 DAP and the stress reached acute level at 35 DAP to 40 DAP [1]. This may also suggest that this stage is a critical physiological period before maturity. 

Due to the lack of understanding of host resistance mechanisms and the markers consistently associated with resistance, use of molecular plant breeding for the development of elite aflatoxin resistant inbred lines is difficult. The two highly expressed genes in susceptible Group 1 included dehydrin DHN1and LEA14-A, while the highly expressed genes in resistant Group 2 included two antifungal plant defensin proteins (γ-thionin, putative hydroxyproline-rich glycoprotein DZ-HRGP), two antioxidant enzymes (putative glutathione peroxidase, glutathione reductase), five signal transduction mediators (putative MAP kinase, MAP kinase phosphatase, putative salt-inducible protein kinase, leucine-rich repeat transmembrane protein kinase, abscisic acid inducible gene), one transmembrane protein (lipid transfer protein), and four stress response proteins (heat shock protein 21, early drought induced protein, multi resistance protein, proline-rich protein family-like). In the drought stressed study, seven genes were up-regulated over 2X in both B73 and Tex6 under drought condition were putative, two anti-bacteria/antifungal plant defensins protein (antifungal zeamatin-like protein, putative CC-NBS-LRR resistance protein MLA13), and five water stress response proteins (LEA14-A, heat shock protein, heat shock protein 22 precursor, proline rich protein and putative aldose reductase). Franco *et al.* [18] reported that γ-thionin has bactericidal activity against Gram-positive and Gram-negative bacteria, and antifungal zeamatin-like protein in maize has been identified as a factor in resistance to aflatoxin contamination in maize [19,20]. Putative CC-NBS-LRR resistance protein MLA13 was a powdery mildew resistance protein in barley [21], and putative hydroxyproline-rich glycoprotein DZ-HRGP was expressed in response to wounding and bacterial, fungal, and viral pathogen infection [22,23]. Glutathione peroxidase is an enzyme family with peroxidase activity whose main biological role is to protect the organism from oxidative damage [24]. MAPKs are major components downstream of receptors or sensors [25,26], and MAPK kinase could increase freezing and salt tolerance in transgenic plants [27]. It is of interest that the higher expression of MAPK gene was found in Group 2 maize lines and Tex6 under drought condition. Higher expression levels of stress response proteins were common in the resistant maize lines, such as heat shock protein and early drought induced protein also has been found in the resistant Group 2 and Tex6 under drought condition. Therefore, the aflatoxin data from field studies and the gene expression patterns of 94 stress-related genes in these maize lines indicate the existence of multiple genes associated with stress tolerance and disease resistance. The genes studied in this research will aid our understanding of maize-*Aspergillus* interactions and other abiotic factors and could contribute to the public candidate gene testing pipeline discussed by Warburton *et al.* [11] in this special issue of Toxins.
